# Nanoporous Co and N-Codoped Carbon Composite Derived from ZIF-67 for High-Performance Lithium-Sulfur Batteries

**DOI:** 10.3390/nano11081910

**Published:** 2021-07-25

**Authors:** Songqiao Niu, Chenchen Hu, Yanyu Liu, Yan Zhao, Fuxing Yin

**Affiliations:** School of Materials Science and Engineering, Hebei University of Technology, Tianjin 300130, China; n2642275274@163.com (S.N.); h15364966537@163.com (C.H.); 18331525609@163.com (Y.L.)

**Keywords:** Li-S batteries, graphitic carbon, ZIF-67

## Abstract

Lithium-sulfur (Li-S) batteries have nice prospects because of their excellent energy density and theoretical specific capacity. However, the dissolution of lithium polysulfides and shuttle effects lead to a low coulombic efficiency and cycle performance of Li-S batteries. Therefore, designing electrode materials that can suppress the shuttle effect and adsorb polysulfides is of great significance. In this work, a Co and N-codoped carbon composite via heating a type of Co-etched zeolitic imidazolate framework-67 (ZIF-67), nanocube precursor, in inert gas is reported as a cathode sulfur carrier material for Li-S batteries. The experimental results show that high-temperature carbonization results in mesoporous structures inside the material which not only provide ion channels for the reaction but also improve the adsorption capacity of polysulfides. Furthermore, the exposed metal Co sites and N atoms can also inhibit the shuttle effect. When the annealing temperature is 600 °C, the sulfur composite exhibits a good cycling stability and rate performance. The cathode showed an improved initial specific capability of 1042 and still maintained 477 mAh g^−1^ at the rate of 1 C (1 C = 1672 mA g^−1^). Furthermore, at 5 C, a stable specific discharge capacity of 608 mAh g^−1^ was obtained.

## 1. Introduction

With the rapid development of electric vehicles and portable electronic equipment, the demand for batteries’ storage and discharge capacity becomes higher. However, lithium-ion batteries, which are currently extremely widely used, can achieve limited improvement in capacity and energy density [1]. In contrast, lithium-sulfur (Li-S) batteries have a much higher theoretical specific capacity (1672 mAh g^−1^) and energy density (2600 Wh kg^−1^) [2,3,4]. Additionally, sulfur is environmentally benign and abundant on the Earth [5,6], making Li-S batteries a potential commercial battery system [7,8,9]. However, such batteries still face various challenges that need to be addressed [10,11]. First, sulfur and lithium sulfide are insulating materials, resulting in poor conductivity which affects the rate performance. Second, the significant difference in the densities of sulfur and lithium sulfide causes large volume changes during cycling, which damages the electrode materials. In addition, the formed discharge intermediate, namely, lithium polysulfide (LiPS), can be dissolved in the electrolyte, which will increase the viscosity of the electrolyte and reduce its conductivity, on the one hand; on the other hand, the LiPS will flow to the anode, which is called the shuttle effect, leading to the formation of lithium sulfide on the anode, reducing the utilization of sulfur. The above phenomena can adversely affect the battery performance, resulting in poor stability and a reduced cycle life [12,13,14,15,16,17].

To address the aforementioned problems, porous carbon-based nanomaterials have been considered as promising candidates as sulfur hosts because of their matrix, large number of pores and large specific surface area, exhibiting good conductivity, sufficient ion transport channels [18] and the adsorption of LiPS to restrict the shuttle effect [19].

An upcoming category of materials, which can be used to obtain porous carbon structures, is the metal–organic framework materials (MOFs) [20]. MOFs are materials consisting of uniform networks formed by the combination of metal ions or ion clusters with organic ligands. One type of zeolitic imidazolate framework, ZIF-67, is a Co-based MOF material that forms an inorganic porous structure with Co-N-C bridges on annealing in a protective atmosphere. The annealed material is essentially a carbon framework doped with N atoms, which can improve the conductivity, surface polarity and adsorption of LiPS, and Co nanoparticles, which can inhibit the shuttle effect by adsorbing LiPS and promoting its decomposition to short-chain Li_2_S/Li_2_S_2_ [21,22]. Based on the different functionalities of the MOF material, ZIF-67 is advantageous as a sulfur-loading electrode of Li-S batteries.

In this work, ZIF-67 was carbonized at high temperature, and the composition and structural differences in the material before and after heat treatment were analyzed. The two materials were used as sulfur-loading electrodes to prepare Li-S batteries, and their performance was studied. The experimental results show that the material changes from a microporous structure to a mesoporous structure after heat treatment, which enhances the storage and adsorption capacity of the material. Secondly, the bare metal sites of the material can catalyze the electrochemical reaction to reduce the loss of battery capacity. In addition, the framework structure of the material changes from organic to graphite, which improves the conductivity of the material, reduces the internal resistance of the batteries and improves the performance of the batteries significantly.

## 2. Experimental Section

### 2.1. Synthesis of ZIF-67 Nanocubes

First, 20 mg of hexadecyl trimethyl ammonium bromide (CTAB) and 730 mg of Co(NO_3_)_2_·6H_2_O were dissolved in 30 mL deionized water to obtain solution A, and 11.3 g of 2-methylimidazole was dissolved in 150 mL deionized water to obtain solution B. Secondly, the above two solutions were mixed and then stirred for 1 h. A uniform, purple-colored mixture was obtained, which was centrifuged to obtain the precipitate and then washed with deionized water twice, and ethanol twice. Finally, the substance was dried for 12–16 h to acquire ZIF-67 nanocubes.

### 2.2. Preparation of Co-NC600

In order to maintain the nanocube shape after heat treatment, the ZIF-67 powder was annealed in a tubular furnace at 300 °C at a heating rate of 5 °C min^−1^, kept for 2 h under Ar flow, then heated at 600 °C for 2 h with the same heating rate and, finally, cooled naturally. The as-obtained black powder was named Co-NC600.

### 2.3. Preparation of S/ZIF-67 and S/Co-NC600

ZIF-67 and Co-NC600 were subjected to the same treatment, described as follows, to prepare the sulfur-hosting matrix materials for the cathode, namely, S/ZIF-67 and S/Co-NC600, respectively. The powder was mixed with sublimated sulfur powder according to a mass ratio of 1:3 and ground for 30 min. Then, a small amount of CS_2_ solution was added to allow the sulfur to be fully dissolved, and the mixture was ground until CS_2_ completely evaporated. The obtained powder was poured into a poly tetra fluoroethylene (PTFE) hydrothermal reactor filled with argon and then sealed. Finally, the reactor was put in a constant temperature drying oven, at 155 °C for 12 h, to yield the required matrix material incorporated with sulfur.

### 2.4. Material Characterization

X-ray diffraction (XRD) patterns were obtained by (Bruker D8 Karlsruhe, Germany) diffractometer equipped with a Cu-Ka radiation source. The structural details of the microstructure were obtained by a field-emission scanning electron microscope (FE-SEM, Hitachi S-4800, Tokyo, Japan) combined with energy-dispersive spectroscopy (EDS) and a high-resolution transmission electron microscope (HRTEM, JEM-2100F, JEOL, Tokyo, Japan). The N_2_ adsorption/desorption analysis was conducted using Micromeritics ASAP 2020 (Norcross, GA, USA), in order to measure the porosity and specific surface area at 77 K. The elements and valence states of the products were analyzed by X-ray photoelectron spectroscopy (XPS, Thermo Fisher Scientific, ESCALAB 250Xi, Waltham, MA, USA). Thermogravimetric analysis (TGA, PerkinElmer, Series 7, Waltham, MA, USA) was carried out to reveal the weight content of sulfur in S/Co-NC600 at a heating rate of 10 °C min^−1^ in Ar flow.

### 2.5. Electrochemical Measurements

CR2032 coin-type cells were used to implement the electrochemical studies, where S/ZIF-67 and S/Co-NC600 were used as cathodes, and lithium foils were used as anodes. The electrolyte employed was 1 M lithium bis (trifluoromethane sulfonyl) imide (LiTFSI) in a mixed solvent which consisted of 1,2-dimethoxyethane (DME) and 1,3-dioxolane (DOL), with a volume ratio of 1:1, containing 1 wt% LiNO_3_. A Celgard 2325 polypropylene membrane was used as the separator.

Electrode slurries of S/ZIF-67 and S/Co-NC600 were produced by mixing S/ZIF-67 or S/Co-NC600, polyvinylidene fluoride (PVDF) binder and conductive carbon black (80%:10%:10%) in N-methyl pyrrolidinone (NMP) solvent. After stirring for 30 min, the slurry was evenly daubed onto Al foil and dried at 60 °C for 12 h. The charge/discharge performance and cycle life of the cells were evaluated on a Neware battery tester between 1.7 and 2.8 V. The cyclic voltammetry (CV) and electrochemical impedance spectroscopy (EIS) measurements were performed using an electrochemical workstation (Princeton Applied Research, Versa STAT4, Princeton, NJ, USA).

## 3. Results and Discussion

**Figure 1:**
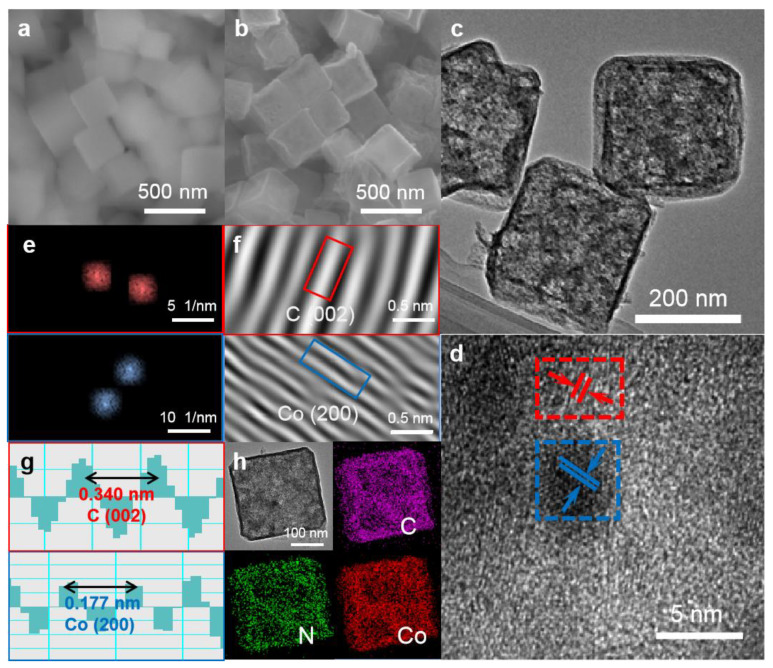
SEM images of (**a**) ZIF-67 and (**b**) Co-NC600; (**c**) TEM image and (**d**) HRTEM image of Co-NC600; (**e**) applied masked points of FFT patterns; (**f**) inverse FFT lattice images of (**e**); (**g**) the lattice spacing contour of Co-NC600; (**h**) elemental mapping of Co-NC600.

Scanning electron microscopy (SEM) images of ZIF-67 and Co-NC600 are shown in Figure 1a,b, respectively. It can be found that the surface of ZIF-67 in Figure 1a is smooth, and there is also a uniform particle size of about 300 nm. The nanocube morphology is different from the conventional dodecahedron because of the addition of CTAB, which changes the growth rate of different surfaces through adsorption on hydrophobic surfaces [23]. In contrast to the ZIF-67 nanocubes, the morphology of Co-NC600 changes slightly, as shown in Figure 1b. The cracking and crosslinking of organic ligands occur due to the heat treatment, which causes a certain degree of depression on the surface of Co-NC600 [24]. Figure 1c shows the TEM image of Co-NC600, which reveals a large number of large-sized pores compared with ZIF-67 shown in Figure S1, possibly due to the splitting of organic ligands. The HRTEM image is shown in Figure 1d, where two types of lattice fringes can be found in the red square and the blue square. The fast Fourier transformation (FFT) patterns of the squares are shown in Figure S2, in which there are many pairs of centrosymmetric light points, proving the existence of the crystal planes. Figure 1e shows that the applied masked points circled in Figure S1 and Figure 1f,g are the inverse FFT lattice images and the lattice spacing contours of Figure 1e. By such figures, it can be measured that two types of layer spacing of 0.340 nm in the red square and 0.177 nm in the blue square of Figure 1d correspond to the (002) crystal face of carbon and the (200) crystal plane of cobalt metal, respectively. It can be found in Figure 1d that Co nanoparticles are inset in the graphitic carbon framework. The high-magnification elemental maps of Co-NC600 are shown in Figure 1h. It can be seen that Co, C and N are evenly distributed onto the nanocubes. Thus, the organic ligands were annealed to form N-doped graphitic carbon frameworks in which the Co nanoparticles were uniformly embedded. The SEM image of S/Co-NC600 and the corresponding elemental mappings in Figure S3 clearly reveal the homogeneous distribution of C, Co and S elements, and that sulfur is well confined in the Co-NC600 nanocubes.

**Figure 2:**
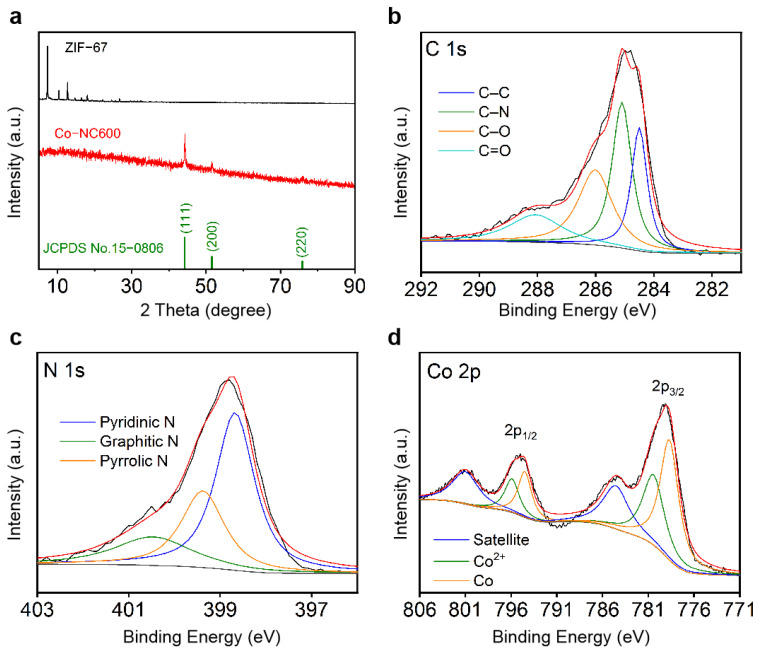
(**a**) XRD pattern of ZIF-67 and Co-NC600; XPS spectra of (**b**) C 1s, (**c**) N 1s and (**d**) Co 2p for Co-NC600.

The XRD patterns of ZIF-67 and Co-NC600 are shown in Figure 2a. In the pattern of ZIF-67, the sharp peak at 7.2° indicates that the prepared ZIF-67 nanocubes have good crystallinity [25]. In the pattern of Co-NC600, there are three distinct peaks at 44.2°, 51.5° and 75.8°, which correspond to the characteristic peaks of the (1 1 1), (2 0 0) and (2 2 0) crystal planes of the metal Co, suggesting that when annealed at 600 °C, the metallic Co particles are exposed. XPS was used to analyze the chemical composition of Co-NC600, whose N, C and Co element XPS spectra are shown in Figure 2b–d. As it is shown in the C 1s spectrum of Figure 2b, it can be found that there are four types of carbon bonds, which correspond to C–C at 284.5 eV, C–N at 285.1 eV, C–O at 286.0 eV and C=O at 288.2 eV. From the high-resolution N 1s spectrum, it can be seen that there are three types of N peaks, i.e., pyridinic N at 398.5 eV, pyrrolic N at 399.2 eV and graphitic N at 400.7 eV [24,26,27]. The vacancy and defect sites due to N doping tend to promote the adsorption of LiPS on the porous material and enhance the reaction kinetics. In addition, the Co 2p spectra of Co-NC600 have four peaks, which, at 779.2 eV and 795.1 eV, correspond to the main peaks of Co 2p_3/2_ and Co 2p_1/2_, respectively, and the other peaks at 784.8 eV and 801.2 eV are the satellite peaks of the above two peaks, respectively. The analysis shows that both main peaks consist of Co (778.6 eV of 2p_3/2_ and 794.4 eV of 2p_1/2_) and Co^2+^ (780.5 eV for 2p_3/2_ and 796.1 eV for 2p_1/2_) peaks. The existence of the Co^2+^ signal is mainly due to the nano-sized Co exposed on the surface of the materials that is partially oxidized by air. The presence of nano-sized transition metals can also enhance the adsorption and catalytic capabilities of the materials.

**Figure 3:**
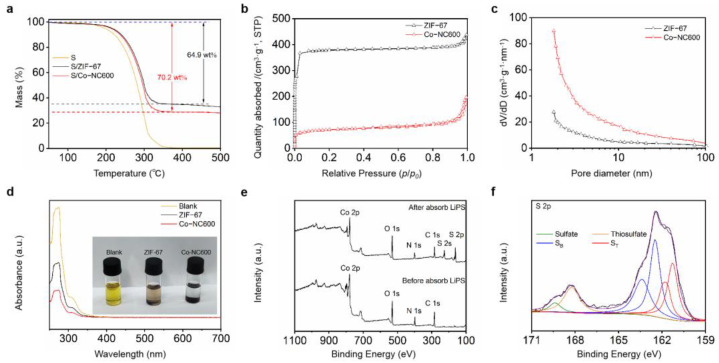
(**a**) TGA curves of S powder and S/Co-NC600; (**b**) N_2_ adsorption/desorption isotherms; (**c**) pore size distributions of ZIF-67 and Co-NC600; (**d**) UV–Vis spectra of Li_2_S_6_ (pristine, with ZIF-67 and Co-NC600), where inset shows the corresponding photograph after 12 h, ZIF-67 and Co-NC600; (**e**) XPS spectra of Co-NC600 before and after Li_2_S_6_ adsorption and (**f**) S 2p spectra of Co-NC600 after Li_2_S_6_ adsorption.

The as-prepared S/Co-NC600 powders were analyzed by TGA, as shown in Figure 3a. When the temperature increases from 150 to 330 °C, the reference sulfur powder sublimates and almost completely disappears, while the mass of S/Co-NC600 reduces to only 29.8%. XPS tests of S/Co-NC600 after annealing were performed to prove that sulfur does not remain in the material during the TGA process, as shown in Figure S4. The above results suggest that the mass proportion of sulfur in the prepared S/Co-NC600 is 70.2%, which reveals that Co-NC600 has a good sulfur loading capacity. Compared with S/Co-NC600, the mass of S/ZIF-67 decreases to 35.1% in the same temperature range, indicating that the mass proportion of sulfur in S/ZIF-67 is 64.9%. It is not difficult to see that Co-NC600 has a better sulfur content than ZIF-67.

Figure 3b shows the N_2_ adsorption/desorption isotherms of ZIF-67 and Co-NC600. The ZIF-67 nanocubes have a high Brunauer-Emmett-Teller (BET) surface area of 1672 m^2^ g^−1^, while, in comparison, the surface area of Co-NC600 is 266 m^2^ g^−1^. After annealing ZIF-67, the BET surface area was significantly reduced, mainly due to the gradual disappearance of the microporous structure in the nanocubes and the formation of a more mesoporous structure, which can be confirmed in Figure 3c. The average pore diameter of Co-NC600 was observed to be 7.67 nm, which was much larger than that of ZIF-67 (1.62 nm).

The ability to capture polysulfides was visualized by a static adsorption test. A typical type of LiPS was used in the test, namely, Li_2_S_6_. Figure 3d shows a pristine Li_2_S_6_ solution, along with the adsorption results of the Li_2_S_6_ solution with ZIF-67 and Co-NC600. After the mixtures were left for 12 h to allow adsorption, the color of the mixture containing Co-NC600 was the lightest, the mixture containing ZIF-67 was lighter than before and the reference showed no color change. The results show that Co-NC600 was more effective at adsorbing Li_2_S_6_ than ZIF-67. UV–Vis spectra further confirmed such a conclusion. As it is shown in Figure 3d, the solution containing Co-NC600 shows the lowest Li_2_S_6_-related absorbance peak. This indicates that the amount of Li_2_S_6_ in the supernatant of the Co-NC600 addition was the lowest, which proves that Co-NC600 had better LiPS adsorption. Co-NC600 is better than ZIF-67 in adsorbing LiPS, firstly because the larger pore structure makes the material more capable of catching LiPS, and secondly because the exposed metal Co nanoparticles in the material interact strongly with LiPS, which anchors LiPS.

Figure 3e shows the XPS spectra of Co-NC600 before and after Li_2_S_6_ adsorption. The appearance of the S 2p peak proves the existence of adsorbed polysulfides. Figure 3f shows the detailed S 2p spectrum in the XPS spectra of Co-NC600 after Li_2_S_6_ adsorption, where the two pairs of peaks at 161.2 and 163.0 eV are attributed to the terminal sulfur (S_T_) and bridging sulfur (S_B_), respectively. The peaks appearing at 169.4 and 168.2 eV illustrate the formation of sulfate and thiosulfate, indicating that the metal Co can react with LiPS chemically, and Co-NC600 has the ability of chemisorption of LiPS.

**Figure 4:**
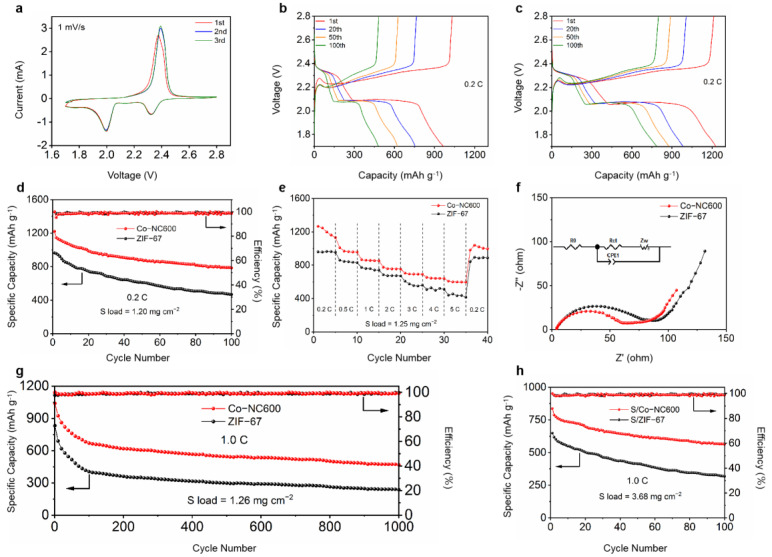
(**a**) CV curves of the cells with the S/Co-NC600 electrode; charge/discharge profiles of (**b**) S/ZIF-67 and (**c**) Co-NC600 electrodes; (**d**) cycling performances of S/ZIF-67 and S/Co-NC600 electrodes; (**e**) rate performances of S/ZIF-67 and S/Co-NC600 electrodes; (**f**) EIS spectra of cells with the cathodes of S/ZIF-67 and S/Co-NC600, where the inset is the equivalent circuit used to fit the impedance spectrum; (**g**) long-term cycling performances of S/ZIF-67 and S/Co-NC600; and (**h**) cycling performances of high-sulfur loading electrodes. The meaning of arrow in d, g and h is to show what the ordinate of the corresponding curve means. The left arrow represents the specific capacity and the right arrow represent efficiency of the curves.

The electrochemical properties of S/Co-NC600 cathodes were tested, which can be seen in Figure 4. The cyclic voltammogram (CV) curves of the S/Co-NC600 electrodes are shown in Figure 4a. The scanning speed was set to 1 mV s^−1^ for three cycles at the range from 1.7 to 2.8 V. The CV shows two related reduction peaks near 2.3 and 2.0 V during discharge, which correspond to the reduction of sulfur to long-chain polysulfide (Li_2_S_x_, 4 ≤ x ≤ 8) and the further reduction of polysulfide to Li_2_S_2_/Li_2_S. In the anodic sweep, the oxidation peaks at 2.4 V correspond to the oxidation of Li_2_S/Li_2_S_2_ to Li_2_S_8_. It is obvious that the three cycle curves almost coincide, which shows that the electrodes have a good electrochemical performance and cycle stability.

Figure 4b,c show the constant current charge/discharge curves of the S/ZIF-67 and S/Co-NC600 electrodes at a rate of 0.2 C (1 C=1672 mA g^−1^), respectively. The initial specific discharge and charge capacities of the S/ZIF-67 electrode are 968 and 1033 mAh g^−1^, and after 100 cycles, the specific capacities decrease to 478 and 492 mAh g^−1^, respectively. In contrast, the S/Co-NC600 electrode has a higher specific capacity, with initial specific discharge and charge capacities of 1237 and 1228 mAh g^−1^, which decrease to 782 and 796 mAh g^−1^ after 100 cycles. The S/Co-NC600 electrode demonstrates a higher specific capacity than the S/ZIF-67 electrode. Similar to other reports of sulfur-based cathodes, the curves have two platforms corresponding to two reduction reactions in all discharge processes of the S/Co-NC600 electrode. During the cycle test, the good overlap of the discharge plateau also shows that the electrode has good stability and reversibility.

The cycling performances of the two types of electrodes were tested at 0.2 C by constant current charge/discharge measurements. In Figure 4d, after 100 cycles, the capacity retention rate is 63%. Such results are better than those of the S/ZIF-67 electrode under the same experimental conditions (the capacity retention rate is 49%), thus demonstrating that S/Co-NC600 has a higher sulfur utilization rate. This is because the metal Co can hold LiPS to inhibit the shuttle effect and reduce the loss of sulfur.

Figure 4e shows the rate capability test of the two electrodes. When the current density increases from 0.2 to 5.0 C, the specific discharge capacity of S/Co-NC600 changes from 1216 to 608 mAh g^−1^. On the other hand, the specific discharge capacity of the S/ZIF-67 electrode changes from 960 to 441 mAh g^−1^. Thus, the S/Co-NC600 electrode showed a better electrochemical performance than the S/ZIF-67 electrode. Moreover, when the current rate is restored to 0.2 C, the specific discharge capacity of the S/Co-NC600 electrode can still reach 1033 mAh g^−1^, indicating that the electrode demonstrated a good recovery. Such properties can be attributed to the structural properties and material composition of Co-NC600. Annealing treatment at high temperatures results in a higher mesopore content, which renders the porous matrix better at sulfur storage and facilitates ion conduction. The exposed metal active sites can also adsorb LiPS to significantly suppress the shuttle effect, which enhances the performance of the batteries.

Figure 4f shows the EIS spectra of the two types of batteries, and the inset is the equivalent circuit, in which R_ct_, Z_w_, constant phase angle element (CPE) and R_0_ stand for the charge transfer resistance, ionic transport resistance, constant phase angle element and other resistance, respectively. The semicircles at the high frequency in the EIS spectra represent R_ct_, while the approximate straight lines at the low frequency correspond to Z_w_ [28]. Compared with the EIS spectra of the S/ZIF-67 electrode, the S/Co-NC600 electrode has a smaller radius semicircle, which means the R_ct_ of the S/Co-NC600 electrode is smaller. The electrode resistance data obtained from the equivalent circuit in Table S1 can also prove such a conclusion. This implies that the charge transfer at the S/Co-NC600 electrode/polysulfide interface is faster, indicating that Co-NC600 efficiently converts LiPS. In addition, the S/Co-NC600 electrode also has a smaller Z_w_, which indicates that the suitable enlarged pore structure can make ion transmission more convenient.

For Li-S battery applications, long-term cycle stability and good capacity retention are critical requirements. The batteries composed of the S/ZIF-67 and S/Co-NC600 electrodes were charged and discharged for 1000 cycles at the rate of 1.0 C. As it is shown in Figure 4g, S/Co-NC600 exhibits an initial specific discharge capacity of 1042 mAh g^−1^ and maintains this at 477 mAh g^−1^ after 1000 cycles. The capacity retention rate is 46%, and the average capacity decay rate per cycle is 0.06%. Compared with the S/Co-NC600 electrode, the S/ZIF-67 electrode has a specific discharge capacity of 827 mAh g^−1^ at the first cycle, and after 1000 cycles, the specific discharge capacity is 237 mAh g^−1^, whose capacity retention rate is 29%. Compared with S/ZIF-67, S/Co-NC600 had a higher specific capacity and better cycling stability.

Co-NC600 has a better electrochemical performance, which benefits from its unique structure. First, Co-NC600 contains more mesopores which are conducive to the adsorption of LiPS, the loading of sulfur and the transport of ions and electrolytes. Secondly, the exposed Co nanoparticles on the carbon frameworks play a very important role in the adsorption of LiPS, which suppresses the shuttle effect. The metal nanoparticles can also greatly enhance the redox reaction kinetics of polysulfides through electrocatalysis. In addition, the doped N atoms induce more active defect sites, which are conducive to a better fixation of polysulfide, thereby effectively suppressing the polysulfide shuttle effect.

Considering the critical importance of sulfur loading of the cathode in practical applications, the cycle performance of the S/Co-NC600 electrode, at a higher sulfur loading, was measured. A coin cell with a sulfur load of 3.68 mg cm^−2^ was assembled and measured for 100 cycles, at the current rate of 1 C. It can be seen in Figure 4h that the initial specific discharge capacity of the battery is 838 mAh g^−1^, which then decreases to 592 mAh g^−1^ after 100 cycles, and the capacity retention rate is 71%. Inevitably, batteries with a higher sulfur load usually suffer from more severe shuttle effects and higher electrochemical polarization, which will lead to a lower sulfur utilization, faster capacity decay and worse cycling stability. Capacity decay was observed in the first three cycles, which may be related to the low sulfur utilization and an increase in the sulfur density. After three cycles, the S/Co-NC600 electrode exhibited high coulombic efficiency, indicating a highly stable performance. The results further reveal that the porous structure and the presence of N atoms and Co nanoparticles were highly conducive to the adsorption and catalysis of polysulfides.

The electrochemical properties of S/Co-NC600 were compared with those of S/ZIF-derived materials reported in the literature, whose results are summarized in Table 1. The proper structured S/Co-NC600 electrode consisting of porous carbon frameworks and plenty of metal nanoparticles shows an excellent initial capacity. Of note, some reported ZIF materials can form regular structures on a larger scale by compounding with conductive carbon materials (CNT, rGO, etc.), which further enhances the ability of sulfur carrying and LiPS adsorption and ultimately improves the performance of Li-S batteries. This provides a new idea for improving the specific capacity and stability of S/Co-NC600 electrodes.

**Figure 5:**
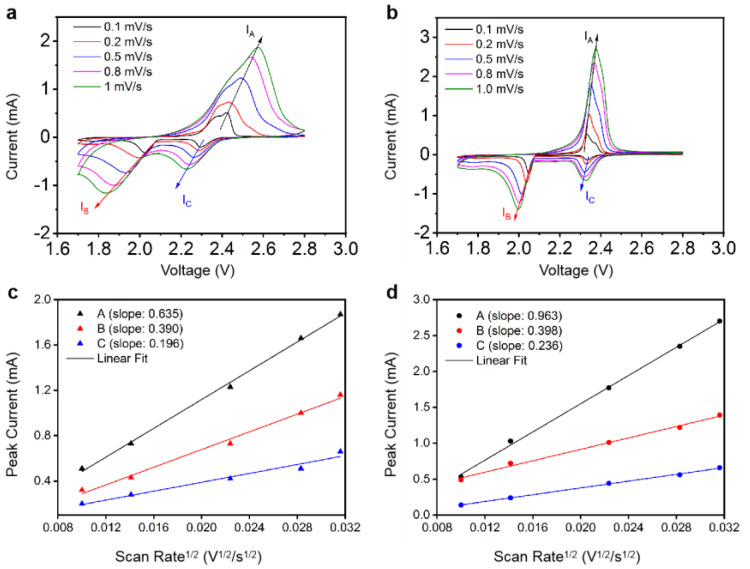
CV curves and corresponding linear fits of the peak currents of Li-S cells with (**a**,**c**) S/ZIF-67 cathode and (**b**,**d**) S/Co-NC600 cathode.

To compare the electrocatalytic activities of Co-NC600 and ZIF-67, the diffusion rates of lithium ions in Li-S batteries with S/Co-NC600 and S/ZIF-67 electrodes were measured by CV tests at various scanning rates from 0.1 to 1 mV s^−1^, as shown in Figure 5. By observing and analyzing the results of Figure 5a,b, it can be found that the peak potential of the S/Co-NC600 electrode changes little with the increase in the scanning rate, and the peak potential of the S/ZIF-67 electrode changes more obviously, which shows that the cells with the S/Co-NC600 electrode have a more stable electrochemical performance. The comparison of the diffusion coefficients of lithium ions is based on the classical Randles–Sevick equation:

I_p_ = 2.69 × 10^5^ n^3/2^ A D_Li_^1/2^ v^1/2^ C_Li_ (at room temperature) (1)

where I_p_ is the peak current, n is the number of charges involved in the reaction, D_Li_ represents the diffusion coefficient of Li in the electrode, v is the scanning rate and C_Li_ represents the concentration of Li^+^ in the electrolyte. According to the formula, there is a linear relationship between I_p_ and v^1/2^, and between I_p_/v^1/2^ and D_Li_^1/2^. When the other parameters are the same, the larger the I_p_/v^1/2^ value, the larger the D_Li_ value, and the stronger the diffusion ability of lithium ions. The linear relationship between I_P_ and v^1/2^ in Figure 5a,b was analyzed, as shown in Figure 5c,d. It can be found that the values of I_p_/v^1/2^ of the S/Co-NC600 electrode (K_A_ = 0.963, K_B_ = 0.398 and K_C_ = 0.236) are obviously larger than those of S/ZIF-67 (K_A_ = 0.635, K_B_ = 0.390 and K_C_ = 0.196). This result shows that the lithium ions in the batteries with S/Co-NC600 electrodes have a better diffusion ability.

The Warburg coefficient in the EIS test can be used to further calculate the lithium ion diffusion coefficient in the battery, as the following equation [36]:

D_Li_ = 1/2 (RT/An^2^F^2^Cσ_w_)^2^ (2)

where R is the universal gas constant (8.314 J mol^−1^ K^−1^), T is the room temperature (298.15 K), A is the area of the electrode surface, n is the number of electron(s) per molecule oxidized F is Faraday’s constant (96,500 C mol^−1^), C is the concentration of lithium ions and σ_w_ is the Warburg coefficient. σ_w_ can be measured by the slope of Z’ (the real part of impedance) and ω (the angular frequency, 2πf)^−1/2^ in the low-frequency region, as shown in Figure S5. Table 2 shows the value of σ_w_ and D_Li_ in the S/ZIF-67 and S/Co-NC600 batteries. Compared with ZIF-67, Co-NC600 has a better ability to promote the diffusion of lithium ions, which can promote the redox process of lithium polysulfide to inhibit the shuttle effects. This is due to the exposed metal Co nanoparticles on the Co-NC600 surface, providing catalytic reaction sites.

## 4. Conclusions

Using ZIF-67 as a template, a Co and N-codoped graphitic carbon material (Co-NC600) was prepared by an annealing process, and both materials were used as cathode materials in Li-S batteries. The microscopic morphology, phase composition and pore structure of the material were analyzed by various measurement methods. Through various analyses, it was found that, compared with ZIF-67, Co-NC600 has conductive carbon frameworks, a larger pore structure and exposed metal nanoparticles. Carbon frameworks can improve the electrical conductivity of the material; an appropriately sized pore structure can improve the abilities of loading sulfur and adsorbing lithium polysulfides; and exposed metal nanoparticles can allow the material to carry on the chemical adsorption of the lithium polysulfides to impede the shuttle effect. Subsequently, the electrochemical performance of different electrodes was systematically studied. This work shows that the performance of the S/Co-NC600 composite electrode is significantly better. The specific discharge capacity in the first cycle at a current rate of 1 C was 1042 mAh g^−1^ and remained at 477 mAh g^−1^ after 1000 cycles. Even at a high current density, at 5.0 C, a stable specific discharge capacity of 608 mAh g^−1^ can be attained.

## Figures and Tables

**Table 1:** The performance of various ZIF-derived materials as sulfur-carrying materials reported in the literature.

Sulfur-Carrier Materials	Current Rate	Initial Discharge Capacity (mAh g^−1^)	Cycle Number	Average Capacity Decay Rate (%)	Reference
Co-NC600	1 C	1042	1000	0.06	In this work
ZIF-8@3DC	1 C/3 C	1098	200/800	0.04 (3 C)	[29]
CNTs/Co_3_S_4_@NC	5 C	850	1000	0.0137	[30]
3DOM ZIF-8	2 C	802	500	0.028	[31]
ZIF-8@rGO	1 C	678	300	-	[32]
ZIF-67-PPy	0.1 C	1093	200	-	[33]
Z-CoS_2_	1 C	~920	1000	0.04	[34]
HZIF/CNT	0.5 C	986	500	0.073	[35]

**Table 2:** The value of the lithium ion diffusion coefficient and Warburg coefficient in the two types of batteries.

Sulfur-Carrier Materials	ZIF-67	Co-NC600
σ_w_ (Ω s^−1/2^)	41.1	5.6
D_Li_ (cm^2^ s^−1^)	1.8 × 10^−15^	1.9 × 10^−13^

## Data Availability

No new data were created or analyzed in this study. Data sharing is not applicable to this article.

## Supplementary Materials

The following are available online at https://www.mdpi.com/article/10.3390/nano11081910/s1, Figure S1. TEM image of ZIF-67 nanocubes; Figure S2. FFT patterns of (a) red square and (b) blue square in the HRTEM image of Co-NC600; Figure S3. Elemental mapping of S/Co-NC600; Figure S4. XPS spectra of S/Co-NC600 after annealing at 350 °C under Ar flow; Figure S5. The liner fit between Z’ and ω^−1/2^ in the batteries with S/ZIF-67 and S/Co-NC600 electrodes; Table S1. Electrode resistance data of S/ZIF-67 and S/Co-NC600 cathodes obtained from the equivalent circuit.
