# Supplementary material for: Nanoporous Co and N-Codoped Carbon Composite Derived from ZIF-67 for High-Performance Lithium-Sulfur Batteries
Source: Nanomaterials (Basel). 2021 Jul 25;11(8):1910. doi: 10.3390/nano11081910 (PMC8400046; doi:10.3390/nano11081910)
Supplement: Supplementary file 1 [file nanomaterials-11-01910-s001.zip › nanomaterials-1286587-SI.pdf]

nanomaterials

MDPI

Supporting information

Nanoporous Co and N-Codoped Carbon Composite Derived from ZIF-67 for High-Performance Lithium-Sulfur Batteries

Songqiao Niu, Chenchen Hu, Yanyu Liu, Yan Zhao * and Fuxing Yin *

School of Materials Science and Engineering, Hebei University of Technology, Tianjin 300130, China; n2642275274@163.com (S.N.); h15364966537@163.com (C.H.); 18331525609@163.com (Y.L.)

* Correspondence: yanzhao1984@hebut.edu.cn (Y.Z.); yinfuxing@hebut.edu.cn (F.Y.)

Citation: Niu, S.; Hu, C.; Liu, Y.; Zhao, Y.; Yin, F. Nanoporous Co and N-Codoped Carbon Composite Derived from ZIF-67 for High-Performance Lithium-Sulfur Batteries. *Nanomaterials* **2021**, *11*, 1910. https://doi.org/10.3390/nano11081910

Academic Editor: Christian M. Julien

Received: 20 June 2021
Accepted: 14 July 2021
Published: 25 July 2021

Publisher's Note: MDPI stays neutral with regard to jurisdictional claims in published maps and institutional affiliations.

Copyright: © 2021 by the authors. Submitted for possible open access publication under the terms and conditions of the Creative Commons Attribution (CC BY) license (http://creativecommons.org/licenses/by/4.0/).

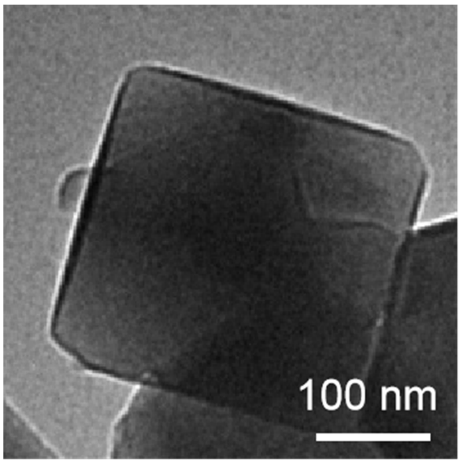

Figure S1. TEM image of ZIF-67 nanocubes.

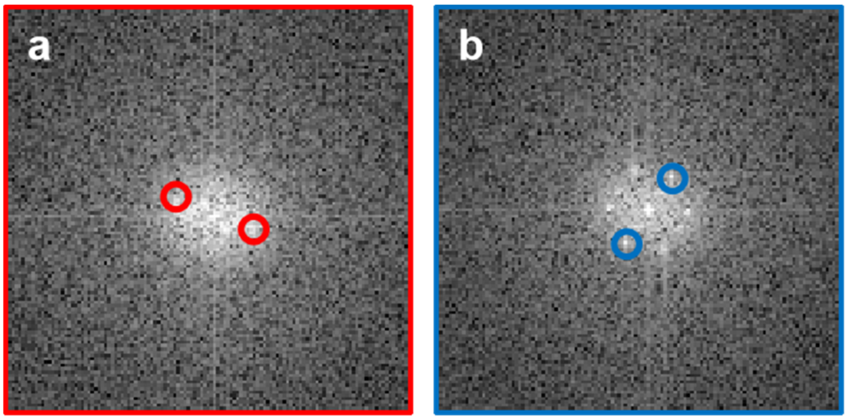

Figure S2. FFT patterns of (a) red square and (b) blue square in the HRTEM image of Co-NC600.

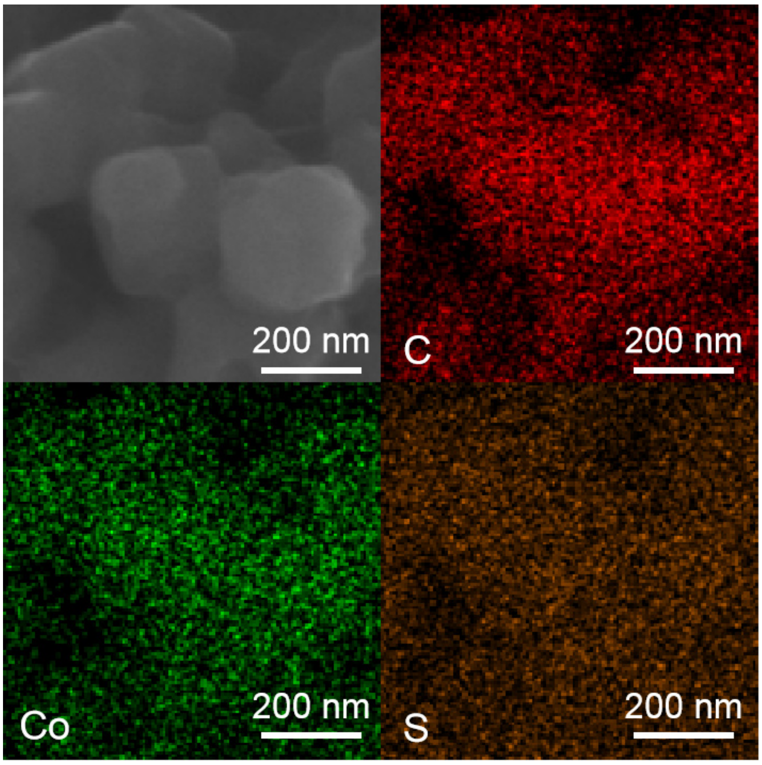

Figure S3. Elemental mapping of S/Co-NC600.

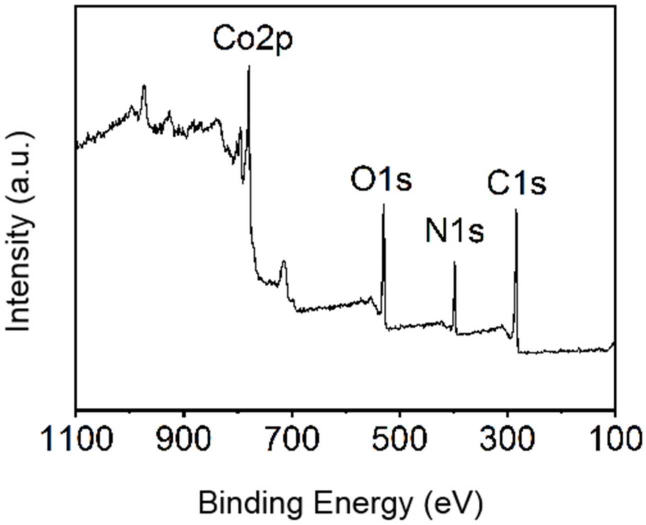

Figure S4. XPS spectra of S/Co-NC600 after annealed at 350 °C under Ar flow.

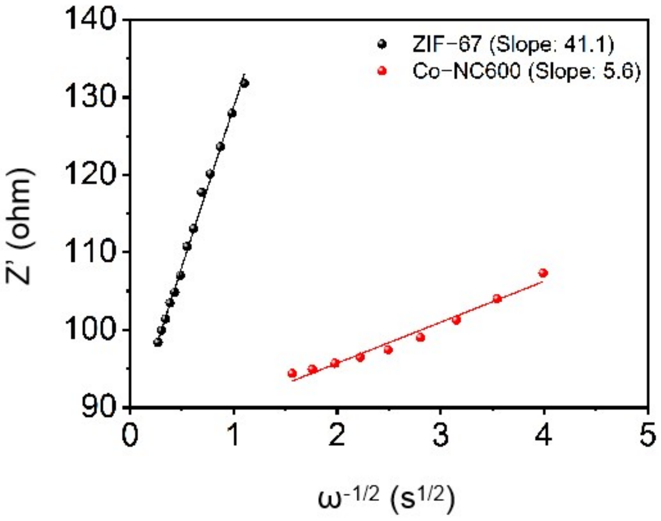

Figure S5. The liner fit between Z′ and $\omega^{-1/2}$ in the batteries with S/ZIF-67 and S/Co-NC600 electrodes.

Table S1. Electrode resistances data of S/ZIF-67 and S/Co-NC600 cathodes obtained from the equivalent circuit.

Sulfur-carrier materials	S/ZIF-67	S/Co-NC600
**R_0 (Ω)**	3.7	3.8
**R_{ct} (Ω)**	72.2	27.9
**Z_w (Ω)**	147.5	60.8
**CPE-P**	0.83	0.72
